# PROTOCOL: Large‐scale food fortification (LSFF) efforts for improving health outcomes in low‐ and middle‐income countries: a systematic review

**DOI:** 10.1002/CL2.204

**Published:** 2018-08-29

**Authors:** Emily C. Keats, Jai Das, Ayesha Siddiqua, Daina Als, Zulfiqar A. Bhutta

## Background

### The problem, condition or issue

Micronutrient malnutrition (MMN) is widely prevalent and significantly associated with the global burden of poverty and disease. In 2000, the World Health Report identified deficiencies in iron, vitamin A, iodine, and zinc as being the most significant risk factors for poor health outcomes globally (WHO, 2000). Together, over one third of the world's population are deficient in these key vitamins and minerals (WHO, 2000). The groups most vulnerable to MMN include pregnant and lactating women and young children (Black, 2001; Black et al., 2008). The WHO states that globally, 190 million preschool children and 19.1 million pregnant women are vitamin A deficient (WHO, 2009), approximately 100 million women of reproductive age (WRA) suffer from iodine deficiency (Leslie, 1991), and 1.62 billion people are anaemic (B Benoist, 2008), though these estimates reflect a relatively large degree of uncertainty. The majority of these individuals live in low‐and middle‐income countries (LMICs) where resources are scarce, diets are not diversified, and health care systems are often not equipped for proper management. The consequences of micronutrient deficiencies by themselves, or in association with overt malnutrition, may relate to both intermediate and long term outcomes. For example: iron‐deficiency anemia is a common consequence of low iron; iodine deficiency can cause stillbirth, miscarriage, and a range of cognitive deficits in neonates; visual impairments and blindness can result from vitamin A deficiency; and congenital anomalies, including neural tube defects (NTDs) are strongly linked to low folic acid intake in pregnancy. While MMN itself has lasting physiological consequences, it also puts an individual at a greater risk for contracting infectious diseases, increasing the severity disease, and experiencing mortality from common infections such as diarrhea, malaria, pneumonia, and measles (Bhutta, 2008). Both maternal and child deaths are a common consequence of MMN. However, adverse outcomes extend beyond health parameters. Economic and social development is hindered as a result of secondary consequences such as lower work productivity and mental and cognitive decline of those who suffer from MMN (Grantham‐McGregor et al., 2007; Hoddinott, 2012; Horton, 2008). Additionally, public health costs are great, inducing a significant burden on both caregivers and health care systems. As such, the international community has become increasingly aware of the importance of dealing with the MMN epidemic.

### The intervention

Large‐scale food fortification (LSFF) refers to the process whereby one or more essential micronutrients are deliberately added to a staple food or condiment during processing in order to improve its nutritional quality. Otherwise referred to as mass fortification, it is a nutrition‐specific intervention that is typically initiated, mandated, and regulated by governments for the purpose of correcting or avoiding micronutrient deficiencies in populations that are at increased risk. Basic commodities, such as flour, oil, salt, sugars, and condiments, are typically chosen as the vehicle for LSFF due to their widespread and regular consumption. Fortificants are defined as the source of micronutrient, while micronutrient premixes refer to that which is blended with the fortificant in order to more feasibly introduce the mixture into the food vehicle. Numerous factors must be taken into consideration throughout planning, including the concentration of the fortificant – such that it is both efficacious and safe, – it's absorptive properties, stability, and how it will affect the sensory qualities of the food vehicle chosen. LSFF is currently promoted as a cost‐effective strategy for reducing physiological and clinical outcomes associated with micronutrient deficiencies, particularly when food is processed centrally and existing technologies and distribution networks can be taken advantage of. This is in contrast to targeted fortification practices that capitalize on specific food vehicles for a particular subset of the population (e.g. infant formula) or home fortification, whereby soluble or crushable tablets, micronutrient powders or micronutrient‐rich spreads are added to foods at the level of the household. Supplementation, usually in the form of pills, capsules and syrups, is another public health intervention aimed at reducing micronutrient deficiencies. Although it has the advantage of supplying an optimal amount of micronutrient per dose, major barriers to the success of this strategy include poor compliance and a lack of supplies, especially in LMICs. To date, the mandatory iodization of salt is the most widely utilized mass fortification measure on a global scale. Reports from UNICEF indicate that in 2013, 75 percent of households globally consumed iodized salt (UNICEF, 2015). Other common examples include vitamin A fortification of sugar and cooking oil, vitamin D‐fortified milk, and iron‐fortified flour. While food fortification has a long history of use in industrialized countries, some LSFF programmes have only recently gained momentum in LMICs.

### How the intervention might work

The purpose of LSFF is to prevent or reverse micronutrient deficiencies by increasing the nutritional content of staple foods that are widely and regularly consumed by the population at risk of micronutrient deficiency. Primary studies conducted in ideal settings suggest that food fortification is likely to be a biologically efficacious strategy: fortificants available in fortified foods have the potential to be absorbed and bioconverted to the active forms necessary to produce the desired health effects in humans (Abrams et al., 2003; Ballot, MacPhail, Bothwell, Gillooly, & Mayet, 1989; Mannar & Gallego, 2002; Muhilal, Permeisih, Idjradinata, Muherdiyantiningsih, & Karyadi, 1988; Solon et al., 2000; Solon et al., 1996; Thuy et al., 2003). However, in real‐world settings, outcomes will depend upon a host of programmatic and contextual factors that will influence the successful implementation of a LSFF program.

**Figure 1:**
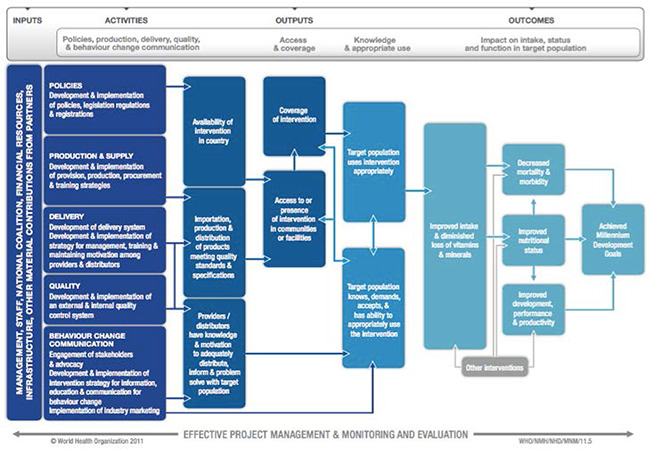
WHO/CDC logic model for micronutrient interventions in public health

In 2009, the Department of Nutrition for Health and Development from the World Health Organization (WHO) and the International Micronutrient Malnutrition Prevention and Control Program from the US Centers for Disease Control and Prevention (CDC) established a working group to develop a standardized logic model for the purpose of implementing successful micronutrient interventions, including LSFF, in public health programs across various contexts (Figure 1) (WHO/CDC, 2011). Though it necessitates adaptation at the local, regional, and national level, this figure outlines the LSFF inputs and activities that should theoretically produce the desired health and nutritional benefits in a population. The following points explain in brief the critical components of a LSFF program.

Prior to implementation, the political climate should be molded such that there is both acceptance and advocacy from leaders. This will create an enabling environment that encourages participation from both the private and industrial sectors. Often, LSFF efforts are controlled through food laws and related practices, which help to specify the logistical elements of a program. These features, such as fortificant type, concentration, and food vehicle, are based upon the defined public health objectives and prevalence of micronutrient deficiencies, population‐level dietary patterns, and usual micronutrient intakes (WHO & FAO, 2006). Feasibility is another critical arm of any food fortification program, and includes the availability of supplies, manufacturers, and distribution channels, as well as the costs required to both implement and sustain the intervention. For the continued availability of adequately fortified foods in a population, quality control and quality assurance mechanisms are critical. Additionally, appropriate marketing and education strategies are essential to communicate the benefits of fortification to consumers such that uptake is sustained. This is an especially important component in the case of voluntary fortification. The latter half of the pathway indicates intermediate outcomes, such as accessibility, awareness, and reach, that can significantly influence LSFF performance overall.

Though the pathway is portrayed as linear, multiple feedback loops exist throughout. The end result, whether positive or negative, will link back to the preceding portion(s) of the pathway such that adjustments can be made where appropriate to obtain maximal benefit. With each of these mechanisms in place, and with the appropriate cross‐talk between them, it is thought that LSFF will have a sustained and beneficial impact among target populations.

### Why it is important to do the review

Food fortification, as a strategy, has been used safely and effectively to prevent micronutrient deficiencies in HICs for more than a century. It is becoming an increasingly attractive investment in LMICs for several reasons, including rapid urbanization and increasing household purchasing power leading to a greater proportion of the population relying on centrally‐processed foods. However, up to this point, evidence to document the true impact of LSFF in LMICs has been limited.

There have been a small number of systematic reviews of efficacy and effectiveness trials attempting to discern the effects of single and multiple micronutrient food fortification on infants and school children (Best et al., 2011; Eichler, Wieser, Ruthemann, & Brugger, 2012; Ramakrishnan, Goldenberg, & Allen, 2011). Two reviews (Best et al., 2011; Eichler et al., 2012; Ramakrishnan et al., 2011) focused on developing countries, while a third (Best et al., 2011) was not restricted by location. When the results are taken together, it was established that LSFF improved micronutrient status, reduced anemia prevalence, and increased linear growth. However, effects on other intermediate and functional outcomes varied depending on the study setting. Another recent review (Pachon, Spohrer, Mei, & Serdula, 2015) specifically examined the effectiveness of government‐supported flour fortification programs on iron status and anemia in children and women of reproductive age. Evidence pertaining to children was, once again, inconclusive. Overall, gaps in this body of knowledge have impeded us from establishing the effects of fortification on childhood morbidity, mortality, and development.

A recent systematic review by Das et al. (Das, Salam, Kumar, & Bhutta, 2013) evaluated the impact of single, dual, or multiple micronutrient fortification of staples, condiments, or processed foods on both women and children's health globally. Results differed between children and women, though both showed improvement of serum micronutrient levels following fortification. Comparable to findings by others, zinc showed no effect on iron absorption of children, though did increase linear growth. Among women, fortification with calcium and vitamin D was shown to have a significant impact among the post‐menopausal age group only. The use of folate significantly decreased the prevalence of congenital abnormalities and, similarly, salt iodization was able to reduce hypothyroidism among women. Again, evidence on morbidity and mortality outcomes in both women and children was lacking, and Das noted that evidence from the developing world was too scarce to comment on the effects of fortification in this setting.

Indeed, although numerous fortification strategies have been shown to be efficacious in many HICs (Das, Kumar, Salam, & Bhutta, 2013; Eichler et al., 2012; T Jiang, 2010), more rigorous and relevant evidence is required from LMICs. Existing reviews are limited to specific subsets of populations and are based on controlled trials only. The current evidence has yet to be coherently analyzed to assess the relevance of findings from programmatic settings or large‐scale effectiveness evaluations in community settings. A more comprehensive review is required – one that takes into account both quantitative (effectiveness) and qualitative (analysis of programs/barriers/lessons) measures, especially in real‐world programmatic settings.

This review will summarize evidence on the impact of LSFF programs for staple foods in LMICs. It will provide a comprehensive synthesis of LSFF effects on health and nutritional status in various contexts. Key recommendations will be succinctly summarized, and dissemination of findings for use in advocacy, policy, and programmatic decision‐making will take place where possible.

## Objectives

The review was commissioned by the Global Alliance for Improved Nutrition (GAIN), who specified that the micronutrients for inclusion in the review should include iron, iodine, folic acid, vitamin A, vitamin D, and calcium, either individually or within multiple micronutrient combinations. Typical staple foods include salt, wheat and corn flour, rice, milk, sugar, and soy sauce.

The review has one main objective: To determine the impact of large‐scale food fortification (LSFF) of staple foods with key micronutrients (iron, folic acid, iodine, vitamin A, calcium, vitamin D), individually or in combination thereof, on primary and secondary health outcomes in low‐ and middle‐income countries (LMICs).

## Methodology

### Criteria for including and excluding studies

#### Types of study designs

Eligible study designs for estimating causal effects include:

- Case‐control studies
- Cross‐sectional studies (including those using instrumental variable designs or statistical matching)
- Discontinuity designs (where eligibility is based on a threshold on an ordinal or continuous variable like a consumption expenditure or poverty index)
- Interrupted time series (ITS) designs
- Longitudinal cohort studies
- Natural experiments
- Serial cross sectional studies where before/after analyses are possible (including panel data studies with multiple observations on the same individuals/groups over time)
- Randomized controlled trials (RCTs)

In order to capture LSFF effectiveness (i.e. impact in a real‐world setting), only national implementation programs or large‐scale trials and observational studies will be considered for inclusion. For trials with multiple arms, only those that meet the eligibility criteria will be included. We will also include data from unpublished studies (including grey literature) to reduce the effects of publication bias.

Efficacy^1^ or proof‐of‐concept studies comparing micronutrient compounds, fortificant concentrations, or food vehicles, will be excluded.

#### Types of participants

Participants will include all residents of low‐ and middle‐income countries (LMICs), as defined by the World Bank Group at the time of the search. While we are aiming to assess males and females of all ages, studies will not be excluded if participants are comprised of only one subset (e.g. children <5 years of age). Where data allows, the review will describe the intervention effect on population subgroups (e.g. urban versus rural populations) in order to consider whether there are issues of equity in access to fortified staple foods.

Studies reporting on populations in high‐income countries will be excluded.

#### Types of interventions

Eligible interventions are large‐scale food fortification (LSFF) programs – the mandatory or voluntary addition of essential micronutrients to widely consumed staple foods or condiments – for the purpose of improving health outcomes of populations. This review will focus specifically on LSFF with iron, vitamin A, iodine, folic acid, calcium, vitamin D, or multiple micronutrients. There are no restrictions regarding duration of exposure to the intervention, whom it is provided by, or the food vehicle that is utilized, pending it is considered a staple food in the context in which it has been implemented.

Excluded interventions provide targeted fortification, home fortification, or biofortification, or use food vehicles that are considered non‐staple foods or condiments, including fortified blended foods and complementary foods.

Comparators may include no treatment (non‐fortified foods), standard care (usual feeding practices), alternate treatment (e.g. diet supplementation), or wait‐list. Author‐defined comparison groups will be used for all analyses.

#### Types of outcome measures

The following is a non‐exhaustive list of outcomes that are considered relevant to our review (see also Table 1). While functional outcomes are more relevant for stakeholders, biochemical outcomes are more often reported, as they are easier to measure. As such, we will include both types of outcomes. Primary outcomes are marked in asterisks.

**Table 1:** Types of outcome categories

**Population**	**Type**						
Perinatal/neonatal	Birth outcomes						
Maternal	Maternal & pregnancy		Birth outcomes			Other (e.g. reproductive health)	
Children <5	Anthropometry	Morbidity	Micronutrient deficiency	Biochemical status	Developmental outcomes	Mortality	Other (specify)
School‐aged children							
Women of reproductive age							
Adults							
Population‐based							
Household	Purchasing		Consumption			Other (specify)	

*Biochemical outcomes:*

Prevalence of micronutrient deficiency or change in micronutrient status, as measured by dietary intake or biomarkers of nutrient status:

*Serum retinol
- Serum folate
- Red blood cell (RBC) folate
- Serum ferritin
- Plasma retinol
- Plasma ferritin
- Haemoglobin concentration
- Urinary iodine concentration
- Serum vitamin D
- Serum phosphate
- Serum calcium
- Serum parathyroid hormone
- Serum zinc.

Studies assessing bioavailability will be excluded.

*Functional health outcomes:*

Deficiency diseases and nutrition disorders where the prevalence or rate is provided before and after LSFF. These include, but are not limited to:

- Acute malnutrition (defined as weight for height)
- Stunting (defined as height for age)
- Underweight (defined as weight for age)
*Anemia
- Hypo/hyperthyroidism
*Goiter
- Night blindness and xerophthalmia
- Adverse pregnancy outcomes
*Neural tube defects (NTDs)
- Neurological impairment
- Cognitive dysfunction
- Bone mass density
- Morbidity
- Mortality.

This review will also highlight any adverse effects of the intervention that have been reported on within included studies. Study eligibility will not be based on outcome reported. However, the quantitative analysis will be restricted to only those studies that report a relevant biochemical or functional outcome in one or more studies.

The selection process will be documented in detail, using a PRISMA flow chart. Any *post‐hoc* decisions that are made regarding the eligibility of a study for inclusion will also be documented within the review. In the case that a study is excluded from the quantitative analysis based on outlier results, sensitivity analyses will be performed.

#### Duration of follow‐up

There are no restrictions regarding duration of follow‐up.

#### Types of settings

All low and middle‐income settings are eligible.

#### Additional inclusion criteria

As a logistical decision based on time/resource constraints, the review will be limited to reports published in English. Studies that report in a language other than English will be excluded. This will be noted as a limitation within the review.

#### Examples of excluded studies

Zimmerman, M.B. et al. *Comparison of the efficacy of wheat‐based snacks fortified with ferrous sulfate, electrolytic iron, or hydrogen‐reduced elemental iron: randomized, double‐blind, controlled trial in Thai women.* Am J Clin Nutr. 2005 Dec:82(6):1276‐82.

Reason for exclusion: efficacy study comparing iron compounds.

Sari, M. et al. *Effect of iron‐fortified candies on the iron status of children aged 4–6 y in East Jakarta, Indonesia.* Am J Clin Nutr. 2001 Jun:73(6):1034–9.

Reason for exclusion: candies are not considered a staple food.

### Search strategy

All appropriate, available electronic reference libraries of indexed medical journals and analytic reviews will be utilized. The search strategy will be run in a total of 15 databases, chosen based on their relevance to the subject material of the review, and guided by the PICO methodology (Table 2):

**Table 2:**
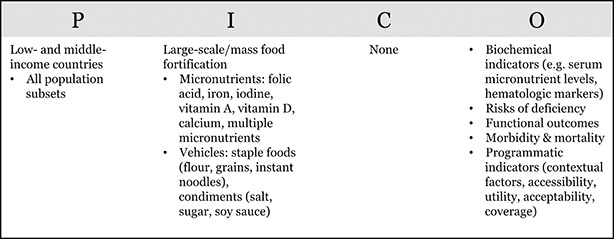
PICO table formulating the review search query

- Medline
- Embase
- CAB Abstracts
- Cochrane Library
- Cumulative Index to Nursing and Allied Health Literature (CINAHL)
- Popline
- Jolis Catalogue
- Jolis Plus
- WHO regional databases (WHOLIS)
- System for Information on Grey Literature in Europe (SIGLE)
- LILACS database for Latin American and Caribbean Health Sciences Literature
- British Library for Development Studies (BLDS)
- IDEAS database for economics and finance research
- Google
- Google Scholar.

The search will be conducted using indexing terms, including medical subject (MeSH) headings and free text words. Guided by our PICO model (table 1), the search brackets fall into 5 categories: staple foods and condiments, key micronutrients, large‐scale/mass food fortification, outcomes, and population. There will be no date restriction on published articles. The search process, including the month/year of search, will be documented for replicability. Details of the search strategy can be found in appendix 1. Manual searches will be conducted within the reference lists of review articles and included primary studies in order to identify any additional sources of information.

Non‐indexed grey literature searches will be conducted primarily in Google and Google Scholar. These may include program descriptions, government reports, conference abstracts, or workshop reports. Grey literature documents will be provided by the Global Alliance for Improved Nutrition (GAIN). We will also search other standard sources of grey literature such as SIGLE/OpenGrey. Additionally, key international nutrition agencies such as The Micronutrient Initiative, will be individually contacted for further information. We will also conduct snowballing of references, and hand‐searching relevant journals and websites as appropriate.

Relevance decisions will be based on two rounds of screening: titles and abstracts, followed by full text. Two independent reviewers will screen both abstracts and full texts in order to identify studies adhering to the inclusion/exclusion criteria. Any disagreements on selection of studies will be resolved by a third, independent reviewer. Quality assessment will also be performed in duplicate for each study.

### Description of methods used in primary research

Because of the inclusion criterion that states that studies must be evaluating nationally implemented fortification programs or large‐scale trials only, many similar methodologies are found within the primary research covered by this review. Most abundant are ITS designs, which examine a specific health outcome in a population, or segment of a population, at multiple time points prior to and following the implementation of a food fortification program. To exemplify these methods, we describe details of eligible studies in the following section.

Hertrampf, E. and Cortes, F. *National food‐fortification program with folic acid in Chile*. Food Nutr Bull. 2008 Jun:29(2 Suppl):S231‐7.

In the year 2000, the Chilean Ministry of Health implemented a National program that mandated the fortification of all wheat flours with 2.2mg/100g folic acid in an attempt to combat neural tube defects (NTDs). This study utilized a hospital‐based prospective time‐series design to examine the prevalence of NTDs in all live and stillbirths during a pre‐fortification (1999–2000) and post‐fortification (2001–2002) period. This hospital‐based surveillance system encompassed nine public hospitals in the city of Santiago, accounting for 60 percent of births in Santiago and 25 percent in all of Chile. A trained staff member was responsible for reviewing all birth documentation, registering and describing NTD. A clinical geneticist reviewed all diagnoses to confirm correct NTD type (anencephaly, spina bifida, or encephalocele). Total prevalence rates were then calculated as total number of NTD per 10,000 births for both time periods.

Using hospital‐based surveillance is a common design when measuring NTD rates. Many other studies pertaining to LSFF use cross‐sectional methods to capture data on other health outcomes, such as anemia. The following study is one such example:

El Hamdouchi, A. et al. *Does flour fortification with electrolytic elemental iron improve the prevalence of iron deficiency anaemia among women in childbearing age and preschool children in Morocco?* Mediterr J Nutr Metab. 2003(6):73–78.

This study examines the prevalence of iron‐deficiency anemia (IDA) in two post‐fortification periods (May 2006 and January 2008) following a wheat flour fortification program with iron in Morocco. The target population consisted of women aged 15–49 years and preschool children aged 2–5 years who were recruited at 38 health centres covering both rural and urban areas of the country. The centres were chosen based on their accessibility to patients, pre‐existing maternal and child health activity, and proximity to a hospital laboratory. To measure serum hemoglobin (Hb), blood was collected from each participant and transported to the laboratory for analysis. Anemia was defined by WHO criteria for preschool children as a Hb level <11.0 g/dL [mild= 10–10.9 g/dL, moderate= 7–9.9 g/dL, severe <7 g/dL]. WHO criteria defined anemia among women of childbearing age as Hb <12.0 g/dL [mild= 10–10.9 g/dL, moderate= 7–9.9 g/dL, severe <7 g/dL]. Mean ± standard deviations were calculated for Hb, examined with unpaired *t* tests and deemed significant if p values <0.05. Study results were displayed as both mean Hb (g/dL) and prevalence of IDA (%), broken down into the varying categories, in order to give a clear picture of any changes that occurred between 2006 and 2008.

Of the methods used in primary research to estimate the causal effects of food fortification, cross‐sectional and longitudinally based interrupted time series designs are common. Some additional methodologies include retrospective before and after designs using hospital administrative or surveillance data, repeated cross‐sectional studies, and controlled or randomized controlled trials using large‐scale cluster sampling methods.

### Criteria for determination of independent findings

We do not anticipate non‐independence of study measures to be problematic within our analyses. As most included studies will consist of before/after designs, estimates will be based on separate populations (e.g. hospital‐based surveillance of NTD rates in live and stillbirths before and after LSFF implementation). If multi‐arm studies are included, intervention groups will be combined or separated into different forest plots, and we will ensure that there is no double counting of participants. If an outcome is reported in several different metrics, we will perform unit conversions in order to pool the data. We do anticipate differences in the types of literature and we will ensure that any analysis will take possible sources of dependency into account by grouping papers into studies/programmes and ensuring that no double counting of evidence takes place when synthesizing across programmes.

### Details of study coding categories

Study data will be abstracted into a standardized data abstraction form that is comprised of a general study information sheet and a quantitative outcome sheet. All data abstraction will be done in duplicate.

Each general study information sheet will include the following:

- General information (author, publication year, language of study, study design)
- Study setting (country, length of study, year(s) of data collection, urban/rural)
- Study population (total study population (N), number and description of participants)
- Intervention (date of LSFF implementation, mandatory/voluntary status, level of intervention (e.g. national or regional), micronutrient(s) of interest, fortificant compound, fortificant concentration, food vehicle chosen, duration of intervention, other micronutrient intervention(s) being utilized by participants)
- Additional notes
- Funding source for program/study
- Quality assessment (see section 3.6)

Some of these variables will be considered as potential moderators (see section 3.7).

Each quantitative outcomes sheet will contain the following information:

- Subgroup
- Subgroup (N)
- Outcome category*
- Outcome definition
- Outcome units
- Outcomes (for intervention & comparison group):
  ○ Outcome measure comparison group (or pre‐fortification)
  ○ Outcome measure treatment group post‐fortification
  ○ Standard deviation
- Effect:
  ○ Effect measure (specify type)
  ○ 95% confidence interval
  ○ P‐value of effect measure
  ○ Standard error or t‐statistic
- Additional notes

*Biochemical and functional outcomes will be further broken down into the following categories: anthropometry, micronutrient deficiency, biochemical status, developmental outcome, morbidity, and mortality.

#### Critical appraisal of studies

Individual studies will be critically appraised according to a set of criteria based on study type, using the Cochrane guidelines. All quality assessment will be done in duplicate to ensure reliability.

For ITS studies, the following standard criteria will be taken into consideration: i) independence of the intervention, ii) pre‐specified shape of the intervention effect, iii) effect of the intervention on data collection, iv) knowledge of the intervention during the study, v) incomplete outcome data, vi) selective outcome reporting, and vii) other risks of bias. For RCTs (including cluster‐RCTs), non‐randomized controlled trials, and controlled before‐after studies, the following criteria will be considered: i) adequate randomization, ii) allocation concealment, iii) similar baseline characteristics and outcome measures between intervention and control groups, iv) incomplete outcome data, v) blinded assessment, vi) study contamination, vii) selective outcome reporting, and viii) any other risks of bias will be considered. Assessment of adjustment for confounding and the potential for reverse causality will be included for quasi‐experimental and observational study types. For uncontrolled before and after studies, careful consideration will be given to historic events/confounding variables and the quality of data collection before and after intervention implementation. These studies will be deemed high risk of bias. Loss to follow up of study participants will be assessed for all studies (>20% loss to follow up is considered a serious limitation). Where a study investigates multiple outcomes, risk of bias due to lack of blinding will be considered separately.

‘Low risk of bias’ studies will demonstrate clear measurement of and control for confounding, including selection bias, where intervention and comparison groups are described adequately and risks of spillovers or contamination are small, and where reporting biases and other sources of bias are unlikely. Studies with ‘medium risk of bias’ will be those where there are threats to validity of the attribution methodology, or there are risks of spillovers or contamination, arising from inadequate description of intervention or comparison groups or possibilities for interaction between groups. ‘High risk of bias studies’ are those where there is evidence of spillovers or contamination to comparison groups from the same communities, and reporting biases are evident. The body of evidence for each pooled outcome will then be assessed for quality using the Child Health Epidemiology Research Group (CHERG) grading system, an extension of the guidelines developed by the Cochrane Collaboration and the Working Group for Grading of Recommendations Assessment, Development and Evaluation (GRADE). CHERG incorporates three categories of criteria for quality assessment: (i) volume and consistency of evidence; (ii) effect size; (iii) strength of statistical evidence, as reflected by the p‐value (Walker et al., 2010). Quality assessment information can be found within the study information sheet of the data abstraction form.

### Statistical procedures and conventions

Outcome data that are not in the form of means and standard deviations for continuous outcomes and proportions or number of events for discrete outcomes will be converted to such, where possible. We will ensure that scales are consistently measured across studies, such that an increase or decrease across the scale will always indicates improvement or deterioration of an indicator. Where data are incomplete or in a form that is unable to be converted, the author will be contacted for clarification. In the event that access to additional data is not possible, the outcome will be excluded from further analysis.

Meta‐analyses will be performed wherever participants, interventions, comparisons, and outcomes are sufficiently similar to ensure a meaningful answer (i.e. across similar PICOs). They will be performed separately per micronutrient (vitamin A, iron, iodine, folic acid, vitamin D, calcium, and multiple micronutrients) and outcome (where pre‐ and post‐fortification outcomes are reported in >1 study). We will analyse randomised and non‐randomised studies with contemporaneous comparison groups separately, pooling only where differences in findings are not statistically significant. We will report separately findings from studies without contemporaneous comparators (e.g. uncontrolled interrupted time series and uncontrolled pretest‐posttest studies). Review Manager Software version 5.3 will be utilized. Approximately correct analysis of cluster‐randomized trials, whereby standard errors will be calculated using information on effective sample size, will be used to correct for any unit of analysis errors, where the original study authors failed to do so. Study effect sizes will be appropriately weighted in pooled analysis to take into account inverse variance. Dichotomous and continuous outcomes will be analysed separately. For dichotomous outcomes, results will be presented as summary risk ratios (RR) or odds ratios (OR) with 95% confidence intervals (CI). Continuous data will be presented as standardised mean differences (SMD) between intervention and comparison groups.

There are several characteristics of included studies that will be retained for examination as potential moderators of study outcomes. These include:

- Study country
- Age and sex of participants
- Socioeconomic status
- Urban/rural residence
- Level of education
- Baseline micronutrient status
- Food vehicle utilized
- Fortificant type
- Concentration of fortificant
- Duration of intervention
- Mandatory or voluntary fortification
- Free distribution/market distribution of fortified food and level of implementation (e.g. national, regional, school‐based)
- Multi‐interventional exposure (e.g. diet supplementation along with food fortification).

Subgroup analyses will be performed according to age group for all outcomes. Age groupings will be determined by data that is available, but we would anticipate the following basic categories: children 6–59 months, children >5 years, adolescents 10–19 years, women of reproductive age (WRA) 15–49 years, men 15–49 years. Along with providing the overall population‐level effect of LSFF, we will separate children, WRA, and men into multiple forest plots to visualize this effect of age and sex. Further subgroup analyses (e.g. by study design) will be conducted where possible in order to assess the potential of confounding by each additional moderator variable. For example, subgroup analysis by food vehicle (e.g. vitamin A fortification of oil versus sugar) may prove to determine important differences in effectiveness of a LSFF program. All subgroup analyses will include reporting of statistical tests of the mean difference between groups examined. Any moderator variables that are identified post hoc will be clearly noted as such within the report.

For the interpretation of results, overall effect estimates that have an associated p‐value <0.05 will be deemed statistically significant. Non‐significant findings will be interpreted and reported appropriately. To address heterogeneity among studies, a random effects model will be used, where the weighting of studies is more balanced. Additionally, between‐study heterogeneity will be examined using the I‐squared and Tau‐squared statistics and a Chi‐square test (where a p‐value <0.1 is considered statistically significant), and through visual inspection of forest plots. Accuracy of numeric data (magnitude and direction of effects in reported studies compared to review) will be checked, and sensitivity analyses will be performed in order to determine the robustness of results, including according to study design and risk of bias assessment. Review conclusions will be based solely on findings from the quantitative synthesis of included studies.

### Treatment of qualitative research

We do not plan to include qualitative research.

## Review authors

**Lead review author:** The lead author is the person who develops and co‐ordinates the review team, discusses and assigns roles for individual members of the review team, liaises with the editorial base and takes responsibility for the on‐going updates of the review.

**Table:** 

**Name:**	Emily C. Keats
Title:	Research Associate
Affiliation:	The Centre for Global Child Health, the Hospital for Sick Children (SickKids)
Address:	686 Bay Street, suite 11.9805
City, State, Province or County:	Toronto, Ontario
Post code:	M5G 0A4
Country:	Canada
Email:	Emily.keats@sickkids.ca
**Co‐authors:**	
**Name:**	Zulfiqar A. Bhutta
Title:	Principal Investigator
Affiliation:	The Centre for Global Child Health, The Hospital for Sick Children (SickKids)
Address:	686 Bay Street, suite 11.9805
City, State, Province or County:	Toronto, ON
Post code:	M5G 0A4
Country:	Canada
Email:	Zulfiqar.bhutta@sickkids.ca
**Names:**	Ayesha Siddiqua, Daina Als
Title:	Research Assistants
Affiliation:	The Centre for Global Child Health, The Hospital for Sick Children (SickKids)
Address:	686 Bay Street, suite 11.9805
City, State, Province or County:	Toronto, ON
Post code:	M5G 0A4
Country:	Canada
Email:	daina.als@sickkids.ca
**Name:**	Jai Das
Title:	Assistant Professor
Affiliation:	The Centre for Excellence in Women and Child Health, Aga Khan University
City, State, Province or County:	Karachi
Post code:	
Country:	Pakistan
Email:	Jai.das@aku.edu

## Roles and responsibilities

- Content: Zulfiqar A. Bhutta
- Systematic review methods: Emily C. Keats, Daina Als, Jai Das
- Statistical analysis: Emily C. Keats, Ayesha Siddiqua, Jai Das
- Information retrieval: All

## Sources of support

This review is funded by the Global Alliance for Improved Nutrition (GAIN).

## Declarations of interest

The Global Alliance for Improved Nutrition (GAIN) has a specific interest in large‐scale food fortification programmes and activities. However, GAIN is not involved in the referee process for this review, which has been managed independently by the Campbell International Development Coordinating Group (IDCG).

## Preliminary timeframe

January 2019

## Plans for updating the review

Dr. Zulfiqar Bhutta would guarantee future updates of the review as contact author.

## AUTHOR DECLARATION

### Authors' responsibilities

By completing this form, you accept responsibility for preparing, maintaining and updating the review in accordance with Campbell Collaboration policy. Campbell will provide as much support as possible to assist with the preparation of the review.

A draft review must be submitted to the relevant Coordinating Group within two years of protocol publication. If drafts are not submitted before the agreed deadlines, or if we are unable to contact you for an extended period, the relevant Coordinating Group has the right to de‐register the title or transfer the title to alternative authors. The Coordinating Group also has the right to de‐register or transfer the title if it does not meet the standards of the Coordinating Group and/or Campbell.

You accept responsibility for maintaining the review in light of new evidence, comments and criticisms, and other developments, and updating the review at least once every five years, or, if requested, transferring responsibility for maintaining the review to others as agreed with the Coordinating Group.

### Publication in the Campbell Library

The support of the Coordinating Group in preparing your review is conditional upon your agreement to publish the protocol, finished review, and subsequent updates in the Campbell Library. Campbell places no restrictions on publication of the findings of a Campbell systematic review in a more abbreviated form as a journal article either before or after the publication of the monograph version in Campbell Systematic Reviews. Some journals, however, have restrictions that preclude publication of findings that have been, or will be, reported elsewhere and authors considering publication in such a journal should be aware of possible conflict with publication of the monograph version in Campbell Systematic Reviews. Publication in a journal after publication or in press status in Campbell Systematic Reviews should acknowledge the Campbell version and include a citation to it. Note that systematic reviews published in Campbell Systematic Reviews and co‐registered with Cochrane may have additional requirements or restrictions for co‐publication. Review authors accept responsibility for meeting any co‐publication requirements.

**I understand the commitment required to undertake a Campbell review, and agree to publish in the Campbell Library. Signed on behalf of the authors**:

**Table:** 

**Form completed by:**	**Date:**

## Search strategy: Medline

### 1. Food

exp Food/ or food*.tw. or exp Food Supply/ or exp Agricultural Crops/ or crop*.tw. or exp Flour/ or flour*.tw. or exp Salts/ or salt*.tw. or exp Fish Products/ or exp Soy Foods or sauce*.tw. or exp Cereals/ or cereal*.tw. or exp Dietary Carbohydrates/ or sugar*.tw. or exp Oryza sativa/ or rice*.tw. or exp Milk/ or milk*.tw. or exp Bread/ or bread.tw. or exp Oils/ or oil*.tw. or exp Beverages/ or beverage*.tw. or exp Yogurt/ or yogurt*.tw. or exp Margarine/ or margarine*.tw. or exp Cheese/ or cheese*.tw. or exp Zea mays/ or maize*.tw. or exp Condiments/ or condiment*.tw. or exp Triticum/ or wheat*.tw. or exp Spices/ or curry powder*.tw. or exp Dietary Fats/ or fat*.tw. or exp Dairy Products/ or dairy.tw.

### 2. Micronutrients

(exp Iron/ or iron.tw. or ferr*.tw. or exp Iodine/ or iod*.tw. or exp Vitamin A/ or “vitamin A”.tw. or exp beta Carotene/ or beta carotene.tw. or exp Ferric Compounds/ or exp Folic Acid/ or folic acid.tw. or folate*.tw. or Ferrous Compounds/ or exp Micronutrients/ or micronutrient*.tw.)

### 3. Fortification

(exp Food, Fortified/ or fortif*.tw. or enrich*.tw.)

### 4. Outcomes

exp Deficiency Diseases/ or deficiency disease*.tw. or exp Nutrition Disorders/ or nutrition disorder*.tw. or exp Infant Nutrition Disorders/ or infant nutrition disorder*.tw. or exp Malnutrition/ or malnutrition.tw. or exp Vitamin A Deficiency/ or vitamin A deficien*.tw. or exp Folic Acid Deficiency/ or folic acid deficienc*.tw. or exp anemia/ or anemia.tw. or anemic.tw. or exp Anemia, Iron‐Deficiency/ or iron‐deficien* anemia.tw. or iron deficien* anemia.tw. or exp Anemia, Hypochromic/ or hypochromic anemia.tw. or micronutrient deficiency.tw. or exp Hemoglobins/ or hemoglobin*.tw. or haemoglobin*.tw.or exp Homocysteine/ or homocysteine.tw. or exp Ferritins/ or ferritin*.tw. or exp Receptors, Transferrin/ or transferrin receptors.tw. or exp Transferrin/ or exp Transferrins/ or transferrin*.tw. or exp Protoporphyrins/ or protoporphyrins.tw. or erythrocyte protophorphyrins.tw. or retinol.tw. or folate.tw. or exp Growth Disorders/ or growth disorder*.tw. or exp Wasting Syndrome/ or wasting syndrome*.tw. or wasting.tw. or stunt*.tw. or underweight.tw. or exp Thinness/ or thin*.tw. or exp Body Mass Index/ or body mass index.tw. or bmi.tw. or exp Body Height/ or body height.tw. or exp Body Weight/ or body weight.tw. or height for age.tw. or weight for age.tw. or weight for height.tw. or mid‐upper arm circumference.tw. or exp Infant, Small for Gestational Age/ or small for gestational age.tw. or exp Anthropometry/ or anthropometr*.tw. or exp Body Size/ or body size.tw. or exp Congenital Abnormalities/ or congenital abnormalit*.tw. or exp Neural Tube Defects/ or neural tube defect*.tw. or NTD*.tw. or exp Spinal Dysraphism/ or spinal dysraphism.tw. or spina bifida.tw. or exp Stillbirth/ or stillbirth*.tw. or exp Congenital Hypothyroidism/ or congenital hypothyroidism.tw. or cretinism.tw. or exp Nervous System Diseases/ or nervous system disease*.tw. or neurological impairment.tw. or exp Cognition Disorders/ or cognit*.tw. or exp Mild Cognitive Impairment/ or cognitive impairment.tw. or exp Night Blindness/ or night blindness.tw. or exp Xerophthalmia/ or xerophthalm*.tw. or exp Hypothyroidism/ or hypothyroid*.tw. or exp Goiter/ or (goiter or goitre).tw. or exp Diarrhea/ or exp Diarrhea, Infantile/ or diarrhea.tw. or exp Pneumonia/ or pneumonia.tw. or exp Malaria/ or malaria.tw. or exp Urinary Tract Infections/ or urinary tract infection*.tw. or UTI.tw. or exp Fever/ or fever*.tw. or exp Morbidity/ or morbidity.tw. or exp Fetal Mortality/ or exp Maternal Mortality/ or exp Child Mortality/ or exp Infant Mortality/ or exp Mortality/ or mortality.tw. or exp Heart Diseases/ or heart disease*.tw. or exp Stroke/ or stroke.tw. or exp Depression/ or depression.tw

### 5. Population

(“developing country” OR “developing countries” OR “developing nation” OR “developing nations” OR “developing population” OR “developing populations” OR “developing world” OR “less developed country” OR “less developed countries” OR “less developed nation” OR “less developed nations” OR “less developed population” OR “less developed populations” OR “less developed world” OR “lesser developed country” OR “lesser developed countries” OR “lesser developed nation” OR “lesser developed nations” OR “lesser developed population” OR “lesser developed populations” OR “lesser developed world” OR “under developed country” OR “under developed countries” OR “under developed nation” OR “under developed nations” OR “under developed population” OR “under developed populations” OR “under developed world” OR “underdeveloped country” OR “underdeveloped countries” OR “underdeveloped nation” OR “underdeveloped nations” OR “underdeveloped population” OR “underdeveloped populations” OR “underdeveloped world” OR “middle income country” OR “middle income countries” OR “middle income nation” OR “middle income nations” OR “middle income population” OR “middle income populations” OR “low income country” OR “low income countries” OR “low income nation” OR “low income nations” OR “low income population” OR “low income populations” OR “lower income country” OR “lower income countries” OR “lower income nation” OR “lower income nations” OR “lower income population” OR “lower income populations” OR “underserved country” OR “underserved countries” OR “underserved nation” OR “underserved nations” OR “underserved population” OR “underserved populations” OR “underserved world” OR “under served country” OR “under served countries” OR “under served nation” OR “under served nations” OR “under served population” OR “under served populations” OR “under served world” OR “deprived country” OR “deprived countries” OR “deprived nation” OR “deprived nations” OR “deprived population” OR “deprived populations” OR “deprived world” OR “poor country” OR “poor countries” OR “poor nation” OR “poor nations” OR “poor population” OR “poor populations” OR “poor world” OR “poorer country” OR “poorer countries” OR “poorer nation” OR “poorer nations” OR “poorer population” OR “poorer populations” OR “poorer world” OR “developing economy” OR “developing economies” OR “less developed economy” OR “less developed economies” OR “lesser developed economy” OR “lesser developed economies” OR “under developed economy” OR “under developed economies” OR “underdeveloped economy” OR “underdeveloped economies” OR “middle income economy” OR “middle income economies” OR “low income economy” OR “low income economies” OR “lower income economy” OR “lower income economies” OR “low gdp” OR “low gnp” OR “low gross domestic” OR “low gross national” OR “lower gdp” OR “lower gnp” OR “lower gross domestic” OR “lower gross national” OR lmic OR lmics OR “third world” OR “lami country” OR “lami countries” OR “transitional country” OR “transitional countries” OR Africa OR Asia OR Caribbean OR West Indies OR South America OR Latin America OR Central America OR Afghanistan OR Albania OR Algeria OR Angola OR Antigua OR Barbuda OR Argentina OR Armenia OR Armenian OR Aruba OR Azerbaijan OR Bangladesh OR Benin OR Byelarus OR Byelorussian OR Belarus OR Belorussian OR Belorussia OR Belize OR Bhutan OR Bolivia OR Bosnia OR Herzegovina OR Hercegovina OR Botswana OR Brazil OR Bulgaria OR Burkina Faso OR Burkina Fasso OR Upper Volta OR Burundi OR Urundi OR Cambodia OR Khmer Republic OR Kampuchea OR Cameroon OR Cameroons OR Cameron OR Camerons OR Cape Verde OR Central African Republic OR Chad OR Chile OR China OR Colombia OR Comoros OR Comoro Islands OR Comores OR Mayotte OR Congo OR Zaire OR Costa Rica OR Cote d'Ivoire OR Ivory Coast OR Cuba OR Cyprus OR Czechoslovakia OR Czech Republic OR Slovakia OR Slovak Republic OR Djibouti OR French Somaliland OR Dominica OR Dominican Republic OR East Timor OR East Timur OR Timor Leste OR Ecuador OR Egypt OR United Arab Republic OR El Salvador OR Eritrea OR Estonia OR Ethiopia OR Fiji OR Gabon OR Gabonese Republic OR Gambia OR Gaza OR Georgia Republic OR Georgian Republic OR Ghana OR Gold Coast OR Greece OR Grenada OR Guatemala OR Guinea OR Guam OR Guiana OR Guyana OR Haiti OR Honduras OR India OR Maldives OR Indonesia OR Iran OR Iraq OR Isle of Man OR Jamaica OR Jordan OR Kazakhstan OR Kazakh OR Kenya OR Kiribati OR Korea OR Kosovo OR Kyrgyzstan OR Kirghizia OR Kyrgyz Republic OR Kirghiz OR Kirgizstan OR “Lao PDR” OR Laos OR Latvia OR Lebanon OR Lesotho OR Basutoland OR Liberia OR Libya OR Lithuania OR Macedonia OR Madagascar OR Malagasy Republic OR Malaysia OR Malaya OR Malay OR Sabah OR Sarawak OR Malawi OR Nyasaland OR Mali OR Malta OR Marshall Islands OR Mauritania OR Mauritius OR Agalega Islands OR Mexico OR Micronesia OR Middle East OR Moldova OR Moldovia OR Moldovian OR Mongolia OR Montenegro OR Morocco OR Ifni OR Mozambique OR Myanmar OR Myanma OR Burma OR Namibia OR Nepal OR Netherlands Antilles OR New Caledonia OR Nicaragua OR Niger OR Nigeria OR Northern Mariana Islands OR Muscat OR Pakistan OR Palau OR Palestine OR Panama OR Paraguay OR Peru OR Philippines OR Philipines OR Phillipines OR Phillippines OR Puerto Rico OR Romania OR Rumania OR Roumania OR Russia OR Russian OR Rwanda OR Ruanda OR Saint Kitts OR St Kitts OR Nevis OR Saint Lucia OR St Lucia OR Saint Vincent OR St Vincent OR Grenadines OR Samoa OR Samoan Islands OR Navigator Island OR Navigator Islands OR Sao Tome OR Senegal OR Serbia OR Montenegro OR Seychelles OR Sierra Leone OR Slovenia OR Sri Lanka OR Ceylon OR Solomon Islands OR Somalia OR Sudan OR Suriname OR Surinam OR Swaziland OR Syria OR Tajikistan OR Tadzhikistan OR Tadjikistan OR Tadzhik OR Tanzania OR Thailand OR Togo OR Togolese Republic OR Tonga OR Tunisia OR Turkey OR Turkmenistan OR Turkmen OR Uganda OR Ukraine OR Uruguay OR USSR OR Soviet Union OR Union of Soviet Socialist Republics OR Uzbekistan OR Uzbek OR Vanuatu OR New Hebrides OR Venezuela OR Vietnam OR Viet Nam OR West Bank OR Yemen OR Yugoslavia OR Zambia OR Zimbabwe OR Rhodesia)

A) 2 and 3
B) 3 and 4
C) 1 and 3
D) 1 and 2 and 3
E) 1 and 2 and 3 and 4
F) OR/ A‐E
G) F and 5
